# Melatonin Nuclear Receptors Mediate Green-and-Blue-Monochromatic-Light-Combinations-Inhibited B Lymphocyte Apoptosis in the Bursa of Chickens via Reducing Oxidative Stress and Nfκb Expression

**DOI:** 10.3390/antiox11040748

**Published:** 2022-04-08

**Authors:** Yijia Zhang, Zixu Wang, Yulan Dong, Jing Cao, Yaoxing Chen

**Affiliations:** Laboratory of Anatomy of Domestic Animals, College of Veterinary Medicine, China Agricultural University, Beijing 100193, China; bs20193050473@cau.edu.cn (Y.Z.); zxwang@cau.edu.cn (Z.W.); ylbcdong@cau.edu.cn (Y.D.); caojing@cau.edu.cn (J.C.)

**Keywords:** monochromatic light combination, melatonin, oxidative stress, nuclear receptor, B lymphocyte, apoptosis, chick

## Abstract

Previous studies found that melatonin modulates a combination of green-and-blue-light-induced B-lymphocyte proliferation via its membrane receptors Mel1a and Mel1c. However, in addition to its membrane-bound receptors, melatonin also functions through binding to nuclear receptors RORα/RORβ/RORγ. In this study, we raised 120 chicks under 400–700 nm white (WW), 660 nm red (RR), 560 nm green (GG) and 480 nm blue light (BB) from P0 to P26. From P27 to P42, half of the chickens in green, blue and red were switched to blue (G→B), green (B→G) and red (R→B), respectively. We used immunohistochemistry, Western blotting, qRT-PCR, Elisa and MTT to investigate the influence of various monochromatic light combinations on the bursal B lymphocyte apoptosis and oxidative stress levels as well as estimate whether melatonin and its nuclear receptors were involved in this process. Consistent with the increase in the plasma melatonin concentration and antioxidant enzyme activity, we observed that G→B significantly decreased the *RORα*, *RORγ* mRNA level, inhibited Bax, Caspase-3 and p-iκb, p-p65 protein expression, increased the IL-10 level and Nrf2, HO-1 protein expression, down-regulated the MDA and pro-inflammatory IL-6, TNF-α and IFN-γ levels in the bursa compared with WW, RR, GG, BB and R→B, respectively. Our in vitro results showed exogenous melatonin supplementation inhibited B-lymphocyte apoptosis, decreased IL-6, TNF-α, IFN-γ and ROS production, down-regulated *RORα*, *RORγ* mRNA level and p-iκb and p-p65 protein expression, whereas it improved the IL-10 level and Nrf2 and the HO-1 protein expression in bursal B lymphocyte. Moreover, these responses were abrogated by RORα agonist SR1078 but were mimicked by RORα antagonist SR3335 or RORγ antagonist GSK2981278. In addition, p65 antagonist BAY reversed RORα/RORγ-mediated G→B-inhibited bursal B lymphocyte apoptosis. Overall, we concluded that melatonin nuclear RORα/RORγ mediates G→B-inhibited bursal B lymphocyte apoptosis via reducing oxidative stress and Nfκb expression.

## 1. Introduction

Birds are sensitive to light because of their highly developed visual systems. In addition to the light intensity and photoperiod, the wavelength of light affects the growth and development [1,2], immune [3], reproduction [4] and productive performance [5] in chicks and hens. Most visible light of different colors is a mixture of three monochromatic lights in proportion. These three monochromatic lights are: 660 nm red light, 560 nm green light and 480 nm blue light. Our lab has shown that, compared to white light, 660 nm red light could result in exacerbating the oxidative stress level [6] and inhibit the proliferation of spleen T lymphocyte [7]. By contrast, the 560 nm green light enhanced the mesor and amplitude of the melatonin and all kinds of the liver clock gene [8] and promoted muscle growth [9], meat quality properties [10] and T/B lymphocyte proliferation activity [6,11] during the early growth stage, while the 480 nm blue light is more effective during the later growth stage of chicks [10,12,13]. Therefore, we suggested that, compared with white light, different monochromatic lights have profound effects on the behavior, physiology, production performance and welfare of poultry. Further research found a combination of green and blue monochromatic light (G→B) effectively enhanced the proliferation activity of the bursal B lymphocyte [14]. These studies suggested that different light wavelengths have important impacts on the lymphocyte development in chickens. In addition to the B lymphocyte proliferation activity, the B lymphocyte apoptosis level, as another important indicator, can reflect bursal B lymphocyte development. On the contrary, whether different monochromatic light combinations will have impacts on bursal B lymphocyte apoptosis is unclear. Previous research reported that, when chicks in red light switched to other monochromatic lights (R→W, R→R, R→G, R→B), the body weight increased slowly, but the body weight in R→B was greater than R→R [9]. This result indicated that blue light can ameliorate the adverse effects of red light on the performance and oxidative stress of chicks. Therefore, we chose the R→B group to compare with G→B and B→G, and to detect the difference in the effects of different monochromatic light combinations on bursal B lymphocytes’ apoptosis.

Melatonin, as one neuroendocrine hormone secreted by the pineal gland, could regulate the function of innate immunity [15] and adaptive immunity [16]. At present, melatonin mainly functions through membrane receptors, such as Mel1a, Mel1b and Mel1c. However, melatonin has the ability to penetrate the cell membrane, and the binding sites of melatonin have been detected in purified spleen and thymus lymphocyte nucleus [17]. Thus, in addition to membrane receptors, melatonin also functions through its nuclear receptor pathway. The melatonin nuclear receptor family consists of three members: RORα, RORβ and RORγ. However, the expression patterns of the three members are tissue-specific. RORα can be expressed in the thymus, skin, kidney, muscle and adipose tissue and can regulate a variety of physiological and pathological processes, including immune response, nervous system development, circadian rhythm and oxidative stress. The Mel–RORα pathway is essential for T lymphocyte proliferation, autoimmune function regulation [18] and anti-inflammatory function [19]. RORβ mainly exists in the central nervous system, and its function is mainly related to the circadian rhythm and the development of the nervous system sensory organs [20]. The expression pattern of RORγ is similar to that of RORα, and it is mainly found in immune cells, muscle, adipose tissue, liver and kidney [21]. RORγ is critical for the development and function of immune cells. Previous studies found that RORγ is a key regulatory factor of the differentiation and development of Th17 cells [22] and ILC3 cells [23]. Moreover, a prior study in our lab reported melatonin nuclear receptors mediate green light-activated T lymphocyte proliferation [7]. However, it is unclear whether the ROR nuclear receptors are involved in the melatonin-dependent inhibition of apoptosis in the bursal B lymphocyte.

In addition to affecting the activity of immune cells, melatonin also plays a role in regulating immune function by disrupting the balance between secretion levels of pro-inflammatory and anti-inflammatory cytokines. When the body is in the chronic inflammatory phase, melatonin can protect the body by inhibiting the secretion of TNF-α, IL-1β and IL-6, which are inducers of inflammation, as well as promoting the secretion of IL-2 and IL-10, which are protective factors that reduce inflammation [24]. The phenomenon that melatonin can enhance the immune response through influencing the concentration of cytokines at the inflammation stage is called “cytoprotective effects”. Moreover, melatonin is also involved in the reduction in oxidative stress levels. Firstly, melatonin itself can be used as a strong antioxidant to directly remove oxygen free radicals generated during normal metabolism [25]. Secondly, melatonin can block the iNOS activity in various tissues and reduce the production of reactive oxygen species [26]. Thirdly, melatonin can improve the expression and enzyme activity of Gsh-Px, GSH-RD, SOD, CAT and other antioxidant enzymes, and improve the antioxidant level of tissues through the above aspects so as to protect lymphocytes from oxidative stress [27].

In the present work, we investigated the influence of various monochromatic light combinations on the bursal B lymphocyte apoptosis, as well as further exploring the mechanisms by which melatonin could exert its effects on B lymphocytes to inhibit their apoptosis as well as which melatonin nuclear receptors are involved in this process.

## 2. Materials and Methods

### 2.1. Animals and Treatments

A total of 120 male Arbor Acre chicks (post-hatching Day 0, Beijing Hua du Breeding Co., Ltd., Beijing, China) were randomly divided into four light treatment groups with 30 replicates (at a temperature of 32 °C in the first week and then kept at a temperature of 30 °C, the relative humidity of 60%). Unlike the 400 to 700 nm white light group (WW), the chicks in red light group were raised under 660 nm monochromatic light by an LED system (RR); the chicks in green light group were raised under 560 nm monochromatic light by an LED system; the chicks in blue light group were raised under 480 nm monochromatic light by an LED system. On the P26 at 23:00, we transferred 15 chicks in 560 nm green light group to 480 nm blue light group (G→B), 15 chicks in 480 nm blue light group to 560 nm green light group (B→G) and 15 chicks in 660 nm red light group to 480 nm blue light group (R→B) for breeding until P42. Therefore, the WW, RR, GG, BB groups (before P27) were changed into WW, RR, GG, BB, G→B, B→G, R→B until P42. For more details, refer to those described previously [14]. The light parameters are shown in Table 1.

### 2.2. Sampling

At P42, all chicks were euthanized, then their blood samples were collected and centrifuged for 30 min at 3000× *g*. Then, the plasma was decanted for melatonin measurement. We randomly selected 5 chicks from each light treatment group for subsequent immunohistochemistry, Western blot analysis and qRT-PCR. Another five chicks from each of the remaining chicks in the seven light treatment groups were selected and used for inflammatory factors, antioxidant capacity assay.

### 2.3. Lymphocyte Proliferation Assay

To determine the mechanism of action of melatonin, B lymphocyte was obtained aseptically from bursa of the G→B group, cultured in RPMI 1640 medium and stimulated with 25 μg/mL LPS and 250 pg/mL melatonin, which were both supplied from Sigma (St. Louis, MO, USA). Then, the B lymphocyte was incubated in the automatic-controlled incubator for 44 h. The temperature in the incubator was kept at 37 °C and the CO_2_ concentration was 5%. We used methyl thiazolyl tetrazolium (MTT) assay and stimulation index to evaluate B lymphocyte proliferative activity. The stimulation index was calculated as the optical density (OD) values in stimulated cells compared to optical density values in unstimulated cells. The optical density was measured by using a microplate reader (Synergy HT; BioTek, Winooski, VT, USA) at 570 nm.

In addition, bursal B lymphocytes of the G→B group were prepared with either RORα antagonist (SR3335, 5 μM, MCE, Weehawken, NJ, USA), RORα agonist (SR1078, 10 μM, MCE, Weehawken, NJ, USA), RORγ antagonist (GSK2981278, 1 μM, MCE, Weehawken, NJ, USA), Nrf2 antagonist (ML385, 5 μM, MCE, Weehawken, NJ, USA), Nfκb antagonist (BAY, 1 μM, MCE, Weehawken, NJ, USA) for 30 min before the addition of LPS and melatonin. After 48 h, each plate of treated cells was collected for determining the mechanism of melatonin action and the inflammatory cytokine. We used 6 wells as replicates in each assay.

### 2.4. Elisa Assay

Plasma (*n* = 5) melatonin were measured using the competitive inhibition Enzyme-linked Immunosorbent Assay Kit (USCN Life Science Inc., Wuhan, China) for melatonin. The detection range of the assay was 4.49–1000 pg/mL, and the intra- and inter assay coefficients of variation were <10% and <12%, respectively. Briefly, according to the manufacturer’s protocol, 50 μL serial dilutions of melatonin standard (1000 pg/mL, 333.33 pg/mL, 111.11 pg/mL, 37.04 pg/mL, 12.35 pg/mL and 0 pg/mL) and samples were incubated with 50 μL Detection Reagent A for 1 h and then 100 μL Detection Reagent B for 30 min at 37 °C. Then, 90 μL Substrate Solution (TMB, 3,3′,5,5′-Tetramethylbenzidine) was micropipetted into each well of the microplate and reacted for 20 min at 37 °C, and the reaction was terminated by the addition of 50 μL stop solution. OD values were immediately measured using an ELISA analyzer (Bio-Rad, Model 680, Hercules, CA, USA) at 450 nm. Each sample was measured in triplicate. We created a standard curve with the log of melatonin concentration of the standard on the *y*-axis and the OD values of the standard on the *x*-axis. The concentration of the sample was calculated according to the standard curve.

Plasma (*n* = 5) GSH-Px, CAT, SOD, T-AOC and MDA levels were measured using commercial kits (Beyotime, Beijing, China). GSH-Px, CAT and SOD are well-known scavenger enzymes that protect cells from oxidative stress. SOD was detected by the xanthine oxidase method, and GSH-Px was determined by the rate at which it was converted to the enzymatic reaction of oxidized glutathione disulfide (GSSG). Those values were expressed as units/mL of plasma. T-AOC was detected by converting Fe^3+^ to Fe^2+^. Those values were expressed as mmol/L of plasma. MDA is responsible for inducing oxidative stress, and it reacts with thiobarbituric acid to form a red complex and is expressed as μmol/L of plasma. These results were detected at specific wavelengths (GSH-Px: 340 nm, CAT: 520 nm, SOD: 450 nm, T-AOC: 593 nm and MDA: 532 nm). Five samples were included in each group, and each sample was tested in triplicate.

The levels of reactive oxygen species (ROS) in the bursal B lymphocyte were detected using a commercial assay kit (Nanjing Jiancheng Co., Ltd., Nanjing, China). The cell concentration was then adjusted to 1 × 10^5^ cells/mL and the cells were loaded with 2′-7′-dichlorofluorescein diacetate (DCFH-DA) (10 μM) for 30 min at 37 °C in the dark. Fluorescence intensity was detected at 502/530 nm (excitation/emission) using a fluorescence microscope reader (Synergy HT; BioTek, Winooski, VT, USA) and expressed as the fluorescent intensity normalized to controls for cultured bursal B lymphocyte. Each sample was assayed three times.

Bursa tissues (*n* = 5) were homogenized in ice-cold PBS (pH = 7.4). The supernatants were then extracted by centrifugation (2000× *g* for 10 min) at 4 °C and stored at −80 °C to allow assay of TNF-α, IFN-γ, IL-6 and IL-10 levels using competitive ELISA (Uscn Life Science, Inc., Wuhan, China). The intra-assay CV was <10%, and the inter-assay CV was <12%. The protein concentration was determined using the BCA protein assay kit (Beyotime, Beijing, China). All tests were performed according to the manufacturer’s instructions. Briefly, according to the manufacturer’s protocol, 100 μL serial dilutions of standard (500 pg/mL, 250 pg/mL, 125 pg/mL, 62.5 pg/mL, 31.2 pg/mL, 15.6 pg/mL, 7.8 pg/mL and 0 pg/mL) and samples were incubated for 60 min at 37 °C. After that, added 100 μL Detection Reagent A for 1 h and then 100 μL Detection Reagent B for 60 min at 37 °C. Then, 90 μL Substrate Solution (TMB, 3,3′,5,5′-Tetramethylbenzidine) was micropipetted into each well of the microplate and reacted for 20 min at 37 °C, and the reaction was terminated by the addition of 50 μL stop solution. OD values were immediately measured using an ELISA analyzer (Bio-Rad, Model 680, Hercules, CA, USA) at 450 nm. Each sample was measured in triplicate. We created a standard curve with the log of inflammatory factors concentration of the standard on the *y*-axis and the OD values of the standard on the *x*-axis. The concentration of the sample was calculated according to the standard curve. The data were expressed as pg/mg.

### 2.5. Immunohistochemical Staining

For immunohistochemical staining, the primary antibodies (rabbit anti–Bcl-2,1:1000, Biorbyt, Cambridge, UK; rabbit anti–Caspase-3, 1:1000, CST, Boston, MA, USA) incubated with the sections overnight at 4 °C and visualized by incubating 0.05% 3,3–diaminobenzidine tetrahydrochloride (DAB, Sigma, St. Louis, MO, USA) and 0.003% hydrogen peroxide. A total of 25 fields were randomly selected in each sample. The integrated optical densities (IODs) of positive cells were measured by using Image–Pro Plus software.

### 2.6. Real-Time Reverse Transcription-Polymerase Chain Reaction (qRT-PCR)

Total RNA was extracted from bursa (*n* = 5) with TRIzol testing agent (CoWin Biotech Co., Inc., Beijing, China). The method was modified according to the standard procedure [28]. In brief, cDNA was reverse-transcribed and amplified using the Revertaid™ first strand cDNA synthesis kit (Fermentas Life Sciences, Burlington, Ontario, Canada). Each 12 μL transcription system included 2 μg total RNA, oligo (dT) 18 primer and nuclease-free water. Each reaction was incubated for 5 min at 65 °C and then mixed with 4 μL 5 × reaction buffer, 1 μL RNase Inhibitor 2 μL 10 mM dNTP mix and 1 μL reverse transcriptase. The mixtures were then incubated for 1 h at 42 °C and then 15 min at 70 °C. Real-time polymerase chain reaction (PCR) was performed with AceQ^®^ qPCRSYBR^®^ Green Master Mix (Q141-02, Vazyme, Nanjing, China). Briefly, 2 μL cDNA was mixed with 10 μL 2 × SYBR Green Master Mix, 1 μL forward primer (20 μM) and 1 μL reverse primer (20 μM) in a final volume of 20 μL per reaction. The PCR amplification protocol was 95 °C for 10 min, 40 cycles of 95 °C for10 s, 57 °C for 30 s and 72 °C for 30 s. Melt curve analyses were performed with the default program of the Light Cycler^®^ 480 (Roche, LightCycler^®^ 480 System, Roche Diagnostics GmbH, Bavaria, Germany). The relative mRNA levels were normalized to GAPDH and calculated using the formula 2^−Ct^. The Ct value was calculated by the formula Ct = Ct target gene –Ct reference gene. Table 2 showed primers sequences of RORα, RORβ, RORγ and GAPDH used in the present research, and each sample was repeated in triplicate.

### 2.7. Western Blot Analysis

The proteins (*n* = 5) of bursa were extracted with RIPA lysis buffer and determined concentration with the bicinchoninic acid (BCA) kit (Beyotime, Shanghai, China). Then, the equal amount of protein in each group was added to the SDS–polyacrylamide gel and transferred onto PVDF membranes and blocked for 1 h using 5% skimmed milk. Subsequently, the primary antibodies, including goat anti–Bax (1:1000, Biorbyt, Cambridge, UK), rabbit anti–Bcl–2 (1:1000, Biorbyt, Cambridge, UK), rabbit anti–Caspase–3 (1:1000, CST, Boston, MA, USA), rabbit anti–Nrf2 (1:1000, Proteintech Group, Inc., Wuhan, China), rabbit anti–HO-1 (1:1000, BIOSS, Beijing, China), rabbit anti–p-iκb (1:1000, Abcam, Cambridge, UK), rabbit anti–p-p65 (1:1000, Abcam, Cambridge, UK) or rabbit anti–β–actin (1:4000; Co Win Biotech Co., Inc., Beijing, China), incubated with the membranes overnight at 4 °C. Then, the membranes were washed with TBST and incubated with horseradish peroxidase–conjugated goat anti–mouse/rabbit IgG (1:8000; Co Win Biotech Co., Inc., Beijing, China) for 2 h. The target bands obtained in the blots were scanned and measured using ImageJ 4.0.2 software (Scion Corp., Frederick, MD, USA) The data were expressed as the IOD of the target bands and compared to the corresponding β–actin values. Repeat the test three times for each sample.

### 2.8. Statistical Analysis

The results of each group were expressed as the mean ± standard error and analyzed using SPSS 25.0 software (SPSS, Chicago, IL, USA). The one-way ANOVA was used to evaluate the effects of different light treatment. The significant differences between seven light treatment groups were considered at *p* < 0.05.

## 3. Results

### 3.1. Caspase-3, Bcl-2, Bax, Protein in the Bursa of Chickens at P42

**Figure 1:**
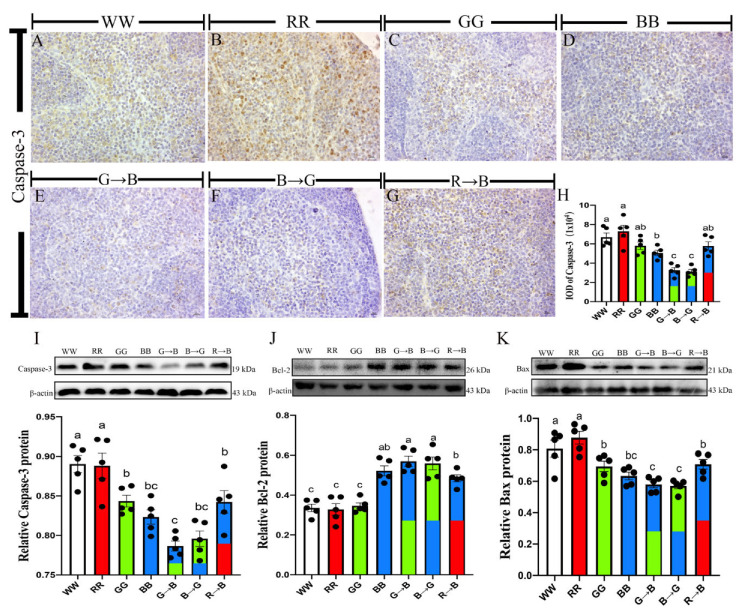
Immunohistochemical staining of Caspase-3 (scale bar = 50μm) in WW (**A**), RR (**B**), GG (**C**), BB (**D**), G→B (**E**), B→G (**F**), R→B (**G**), IOD of Caspase-3 positive cells (**H**), Caspase-3 protein (**I**), Bcl-2 protein (**J**), Bax protein (**K**) in the bursa at P42. The results of each group were expressed as the mean ± standard error. Differences between seven light treatment groups were evaluated by one-way ANOVA, and values with no common letters differ significantly (*p* < 0.05).

As shown in Figure 1A–H, the IOD of Caspase-3 positive cells in bursa was 55.19–123.98% lower in G→B than in WW, RR, GG, BB and R→B (*p* < 0.001). There was no significant difference between G→B and B→G (*p* > 0.05), but G→B was lower than B→G by 4.30%. Similarly, the Western blot analysis showed that the expression of Caspase-3 in the bursa was 7.05–13.13% lower in G→B than WW, RR, GG and R→B (Figure 1I, *p* = 0.000–0.025). In addition, the Caspase-3 protein level of G→B was lower by 1.13–4.62% than that of BB and B→G, but there was no significant difference among G→B, BB and B→G (*p* > 0.05). Next, we tested the expression of Bcl-2 protein, which plays a crucial role in inhibiting cell apoptosis. As shown in Figure 1J, the level of Bcl-2 protein in G→B was 17.66–73.61% higher than WW, RR, GG and R→B (*p* = 0.000–0.017). However, there was no significant difference between G→B, B→G and BB (*p* > 0.05), but G→B was higher than B→G and BB by 1.90–9.12%. G→B also significantly decreased the protein expression of Bax (9.38–51.27%, *p* = 0.000–0.012) and the ratio of Bax/Bcl-2 (18.70–169.47%, *p* < 0.001) compared with WW, RR, GG and R→B, respectively. In contrast to G→B, RR decreased the expression of Bcl-2 protein and increased the expression of Bax and Caspase-3 protein. These results indicated that G→B could inhibit bursal B lymphocyte apoptosis, and RR has the opposite results.

### 3.2. Plasma GSH-Px, CAT, SOD, T-AOC, MDA and Bursal IL-6, TNF-α, IFN-γ, IL-10 Concentration in Chickens at P42

**Figure 2:**
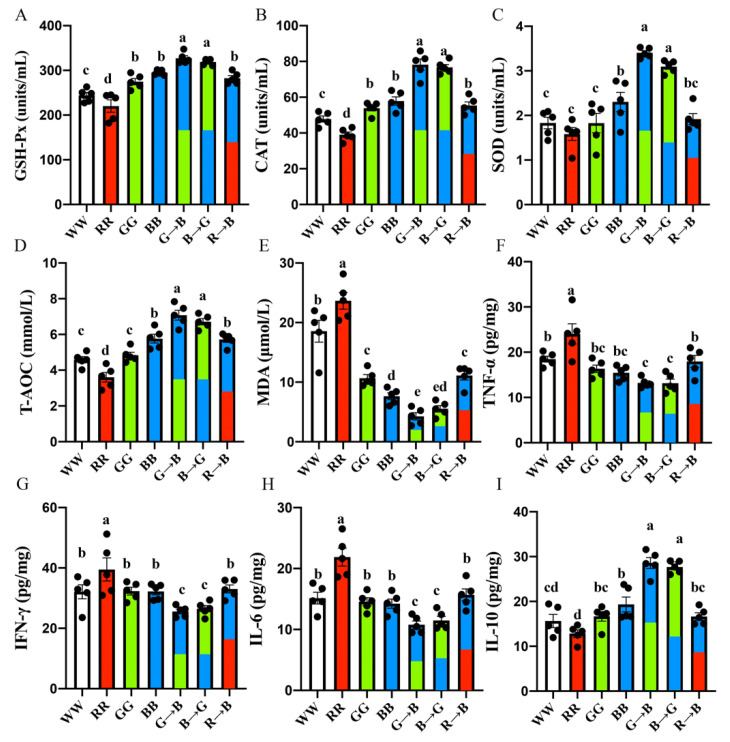
Plasma GSH-Px (**A**), CAT (**B**), SOD (**C**), T-AOC (**D**), MDA level (**E**), bursal TNF-α (**F**), IFN-γ (**G**), IL-6 (**H**), IL-10 (**I**) level in the chick at P42. The results of each group were expressed as the mean ± standard error. Differences between seven light treatment groups were evaluated by one-way ANOVA, and values with no common letters differ significantly (*p* < 0.05).

Then, to evaluate the impact of different light wavelengths upon the oxidative stress level, we determined the change in five antioxidant indices in plasma. As shown in Figure 2A–E, G→B significantly increased the GSH-Px (10.83–48.51%, *p* = 0.000–0.004), CAT (35.07–100.47%, *p* = 0.000), SOD (47.83–115.30%, *p* = 0.000) and T-AOC (22.79–96.45%, *p* = 0.000) levels and decreased the plasma lipid metabolite and MDA levels (78.71–453.59%, *p* = 0.000–0.023) compared with WW, RR, GG, BB and R→B. There was no significant difference between G→B and B→G (*p* > 0.05), but the antioxidant enzymes and T-AOC were higher in G→B than in B→G by 1.93–10.01%, and the MDA level was lower in G→B than in B→G by 29.39%.

In addition, as shown in Figure 2F, G→B significantly reduced the levels of pro-inflammatory cytokine TNF-α (16.89–82.10%, *p* = 0.001–0.007) in the bursa compared with WW, RR and R→B. There was no significant difference between G→B, B→G, BB and GG (*p* > 0.05), but the TNF-α level was lower in G→B than in BB and GG by 16.89–23.73%. Consistent with this result, the IFN-γ and IL-6 levels in the bursa of G→B chicks were significantly lower than WW, RR, GG, BB and R→B by (25.89–54.24%, *p* = 0.000–0.022) and (32.05–103.00%, *p* = 0.000–0.012). Additionally, there was no significant difference between G→B and B→G. However, RR increased the levels of IL-6, TNF-α, and IFN-γ in the bursa compared with WW, GG, BB, G→B, B→G and R→B (*p* = 0.000–0.014). On the contrary, the level of anti-inflammatory factor IL-10 was the highest in G→B but had the lowest level in RR. The IL-10 level was higher in G→B than in WW, RR, GG, BB and R→B (47.72–122.58%, *p* < 0.001) but had no difference between G→B and B→G. In the meanwhile, the GSH-Px, CAT, SOD and T-AOC were the lowest in RR. Therefore, these results suggested that G→B can significantly reduce the oxidative stress level, while RR can significantly aggravate the level of oxidative stress.

### 3.3. Plasma Melatonin Concentration and Melatonin Nuclear Expression in the Bursa of Chickens at P42

First, we explored the influences of various monochromatic light combinations on the plasma melatonin concentration of chicks at P42. As shown in Figure 2A, the plasma melatonin concentration was higher in G→B than in WW, RR, GG, BB and R→B by 6.34–82.26% (*p* = 0.000–0.300). There was no significant difference between G→B and B→G (*p* > 0.05), but G→B was higher than that of B→G by 6.34%. Additionally, there was a strong negative correlation between the melatonin concentration in the plasma and the pro-apoptosis protein expression of Bax (r = −0.97, *p* < 0.001) and Caspase-3 (r = −0.99, *p* < 0.001).

**Figure 3:**
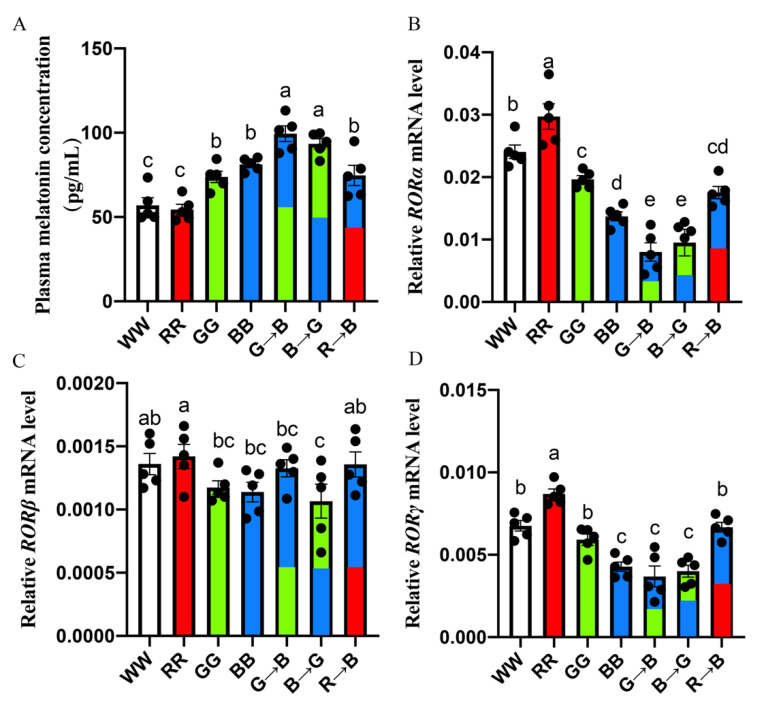
Plasma melatonin concentration (**A**), *RORα* mRNA level (**B**), *RORβ* mRNA level (**C**), *RORγ* mRNA level (**D**) in the bursa at P42. The results of each group were expressed as the mean ± standard error. Differences between seven light treatment groups were evaluated by one-way ANOVA, and values with no common letters differ significantly (*p* < 0.05).

In addition, as shown in Figure 3B, G→B significantly decreased the bursal *RORα* mRNA level (71.58–271.35%, *p* = 0.000–0.008) compared with WW, RR, GG, BB and R→B, and there was no significant difference between G→B and B→G. Similarly, as shown in Figure 3D, G→B significantly decreased the bursal *RORγ* mRNA level (15.85–135.69%, *p* = 0.000) compared with WW, RR, GG and R→B. However, the *RORβ* mRNA level in the bursa had no significant difference between WW, RR, GG, BB, G→B, B→G and R→B (*p* > 0.05). In addition, the Pearson’s correlation analysis showed that the *RORα* mRNA (r = −0.98, *p* < 0.001) and *RORγ* mRNA levels (r = −0.92, *p* = 0.003) were negatively correlated with the plasma melatonin concentration. However, there was no significant correlation between the *RORβ* mRNA level and plasma melatonin concentration (*p* > 0.05). Therefore, these results suggested that melatonin nuclear receptors RORα and RORγ may play important roles in mediating G→B-inhibited bursal B lymphocyte proliferation.

### 3.4. Nrf2, HO-1, p-iκb, p-p65 Protein Level in the Bursa of Chickens at P42

**Figure 4:**
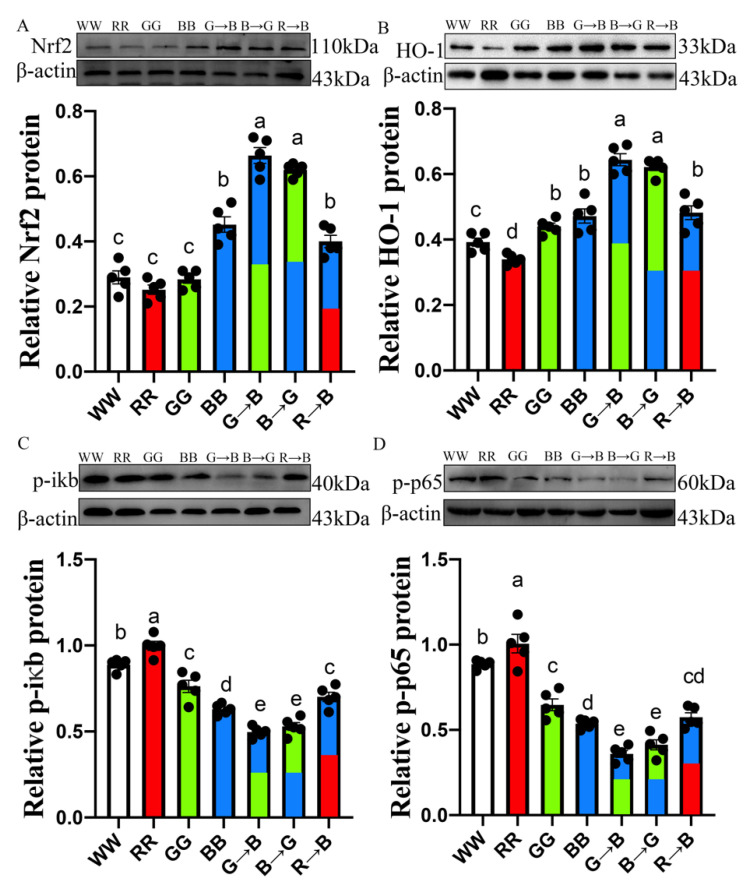
Nrf2 (**A**), HO-1 (**B**), p-iκb (**C**), p-p65 (**D**) protein level in bursa at P42. The results of each group were expressed as the mean ± standard error. Differences between seven light treatment groups were evaluated by one-way ANOVA, and values with no common letters differ significantly (*p* < 0.05).

In order to explore the mechanism by which G→B-inhibited bursal B lymphocyte apoptosis, we investigated the expression of the Nrf2/HO-1 signaling pathway and Nfκb-signaling-pathway-related proteins in the bursa of chickens. As shown in Figure 4A,B, the Nrf2 and HO-1 protein levels of the bursa in G→B were significant higher by 46.90–163.49% (Nrf2, *p* < 0.001) and 36.44–89.41% (HO-1, *p* < 0.001) than WW, RR, GG, BB and R→B, while the p-iκb and p-p65 protein levels of the bursa in G→B were significant lower by 26.54–100.81% (p-iκb, *p* < 0.001) and 48.98–179.41% (p-p65, *p* = 0.000–0.007) than WW, RR, GG, BB and R→B. However, there were no significant differences between G→B and B→G (*p* > 0.05), but the Nrf2 and HO-1 protein levels of the bursa were higher in G→B than in B→G by 3.54–7.10%, whereas the p-iκb and p-p65 protein levels in G→B were significant lower by 6.52–14.59% than B→G. These results indicated that Nrf2, HO-1, p-iκb and p-p65 proteins may play important roles in G→B-inhibited bursal B lymphocyte apoptosis.

### 3.5. Melatonin Nuclear Receptors RORα/RORγ-Mediated G→B-Inhibited B Lymphocyte Apoptosis in the Bursa of Chickens via Nrf2/HO-1 and Nfκb Pathway

**Figure 5:**
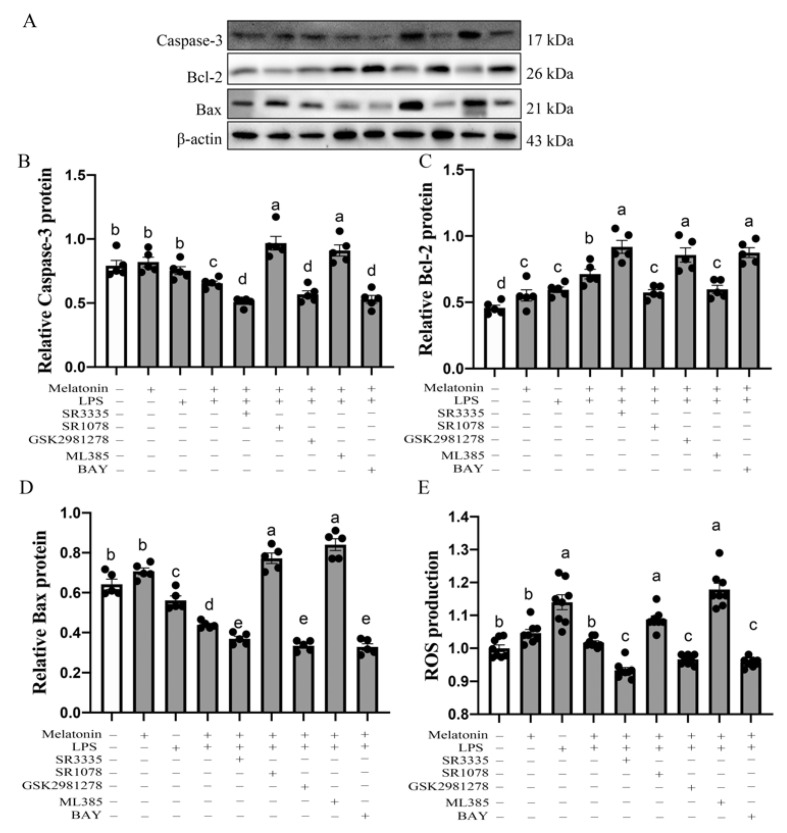
Effects of RORα antagonists, RORα agonist, RORγ antagonist, Nrf2 antagonists and p65 antagonists on Caspase-3 protein (**A**,**B**), Bcl-2 protein (**C**), Bax protein (**D**), ROS level (**E**) of bursal B-lymphocyte in response to LPS in the G→B group. SR3335 is a RORα antagonist; SR1078 is a RORα agonist; GSK1981278 is a RORγ antagonist; ML385 is an NRF2 antagonist; BAY is a p65 antagonist. Differences between seven light treatment groups were evaluated by one-way ANOVA, and values with no common letters differ significantly (*p* < 0.05).

Next, we further verified whether exogenous melatonin could mediate G→B-inhibited bursal B-lymphocytes apoptosis in vitro. We isolated aseptically the bursa B lymphocytes from G→B and detected the expression of apoptosis-related proteins to determine the effect of melatonin on B-lymphocyte apoptosis after 44 h of culture. As shown in Figure 5A–D, LPS + melatonin could significantly increase the anti-apoptosis protein Bcl-2 expression (*p* < 0.001) but significantly decreased the pro-apoptosis protein Bax (*p* < 0.001) and Caspase-3 (*p* = 0.009) levels compared with the control group, respectively. In addition, as shown in Figure 5E, exogenous melatonin supplement could down-regulate ROS production in B lymphocyte compared with the LPS group (*p* < 0.001). To determine the involvement of melatonin nuclear receptors RORα and RORγ on B lymphocyte apoptosis, primary cultures were pretreated with SR3335 (an antagonist of RORα), SR1078 (an agonist of RORα) and GSK2981278 (an antagonist of RORγ). After the pretreatment of B lymphocyte with RORα agonist SR1078, the Western blotting results showed that SR1078 markedly inhibited melatonin-induced Bcl-2 protein expression up-regulation (*p* = 0.011), promoted Bax (*p* < 0.001) and Caspase-3 (*p* < 0.001) protein down-regulation and induced ROS production (*p* = 0.000). In contrast, in SR3335 + LPS + melatonin or GSK2981278 + LPS + melatonin-treated B lymphocyte, the Bcl-2 protein was 28.78–20.17% (*p* = 0.000–0.009) higher than in the LPS + melatonin group, while the Bax, Caspase-3 and ROS level was 18.83–31.32% (Bax, *p* = 0.000–0.021), 15.22–28.75% (Caspase-3, *p* < 0.001) and 5.55–9.26% (ROS, *p* = 0.000–0.005) lower than in the LPS + melatonin group, respectively. Similar to SR3335 and GSK2981278 pretreatment, Nrf2 antagonist ML385 could also markedly reduce the Bcl-2 protein level (*p* = 0.033) and enhance the Bax (*p* < 0.001), Caspase-3 protein (*p* < 0.001) and ROS level (*p* = 0.001). However, when treating bursal B lymphocyte with p65 antagonist BAY, the protein expression of Bax, Caspase-3 and Bcl-2 were opposite of ML385. These results suggested that melatonin mediates G→B-inhibited bursal B lymphocyte apoptosis through nuclear receptor RORα/RORγ and the Nrf2/HO-1 and Nfκb signaling pathway. 

**Figure 6:**
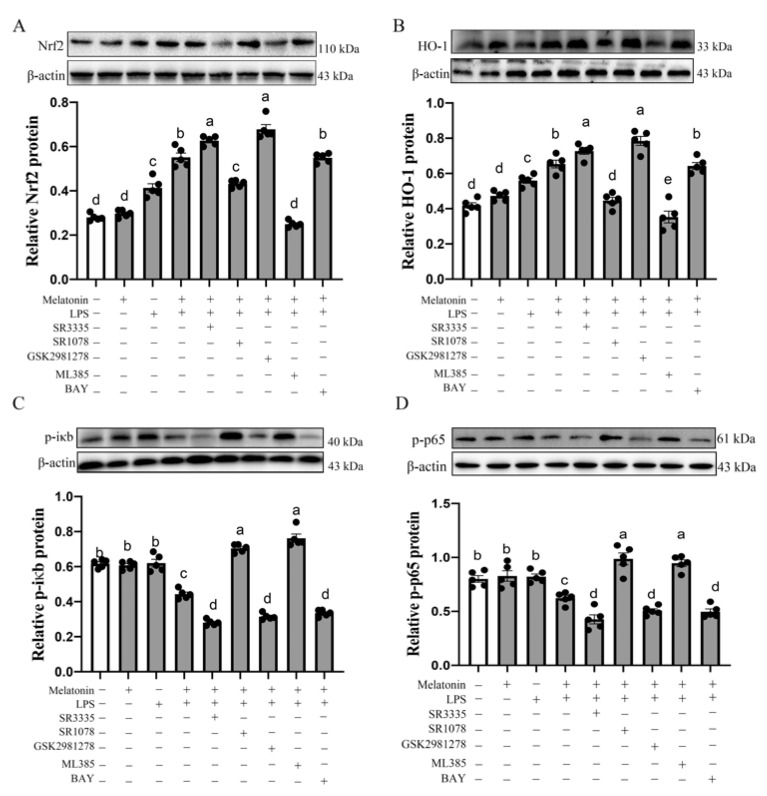
Effects of RORα antagonists, RORα agonist, RORγ antagonist, Nrf2 antagonists and p65 antagonists on Nrf2 (**A**), HO-1 (**B**), p-iκb (**C**), p-p65 (**D**) protein level of bursal B-lymphocyte in response to LPS in the G→B group. SR3335 is a RORα antagonist; SR1078 is a RORα agonist; GSK1981278 is a RORγ antagonist; ML385 is an NRF2 antagonist; BAY is a p65 antagonist. Differences between seven light treatment groups were evaluated by one-way ANOVA, and values with no common letters differ significantly (*p* < 0.05).

To further clarify the respective roles of melatonin nuclear receptors in the Nrf2/HO-1 and Nfκb pathway, we pretreated B lymphocyte with melatonin nuclear receptor antagonist. As shown in Figure 6A,B, the observed melatonin-induced up-regulation in the Nrf2 and HO-1 protein was abrogated by RORα antagonist SR3335 or RORγ antagonist GSK1981278 (*p* = 0.000–0.017). Additionally, melatonin-induced down-regulation of p-iκb and p-p65 protein was also abrogated by the RORα antagonist SR3335 or RORγ antagonist GSK2981278 (*p* = 0.000–0.025). However, the protein levels of Nrf2 and HO-1 in the LPS + melatonin + SR1078 group were significantly decreased compared with the LPS + melatonin group (*p* < 0.001), and the p-iκb and p-p65 protein expression levels in the “LPS+ melatonin +SR1078” group were significantly higher than those in the “LPS + melatonin” group (*p* < 0.001).

Next, we explored further the relationship between the Nrf2/HO-1 and Nfκb pathway in melatonin-inhibited B lymphocyte apoptosis. As shown in Figure 6C,D, the LPS + melatonin + ML385 could significantly increase the p-iκb protein expression in the cytoplasm and p-p65 protein expression in the nuclear receptor compared with the LPS + melatonin group (*p* < 0.001). However, the co-addition of p65 antagonist with LPS and melatonin had no significant effect on the expression of Nrf2 and HO-1 protein level (*p* > 0.05). Therefore, these results suggested that melatonin activates the downstream Nrf2/HO-1 signaling pathway by binding RORα and RORγ receptors, and then the activated Nrf2/HO-1 signaling pathway leads to the activity of p-iκb and p-p65 down-regulation, thereby inhibiting B lymphocyte apoptosis.

**Figure 7:**
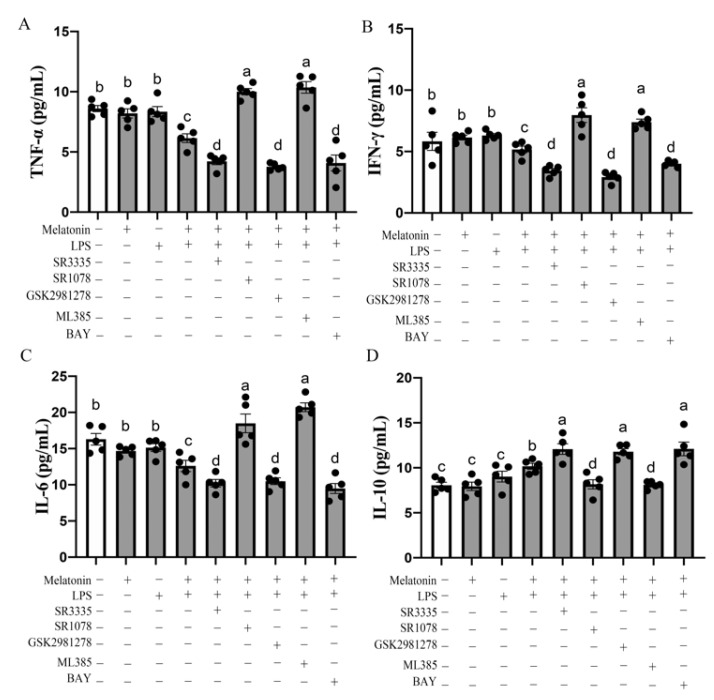
Effects of RORα antagonists, RORα agonist, RORγ antagonist, Nrf2 antagonists and p65 antagonists on pro-inflammatory cytokine TNF-α (**A**), IFN-γ (**B**), IL-6 (**C**), anti-inflammatory cytokine IL-10 (**D**) level of bursal B-lymphocyte in response to LPS in the G→B group. SR3335 is a RORα antagonist; SR1078 is a RORα agonist; GSK1981278 is a RORγ antagonist; ML385 is an NRF2 antagonist; BAY is a p65 antagonist. Differences between seven light treatment groups were evaluated by one-way ANOVA, and values with no common letters differ significantly (*p* < 0.05).

As shown in Figure 7A,B, the pretreatment of B lymphocyte with melatonin nuclear receptor RORα antagonist SR3335 or RORγ antagonist GSK2981278 significantly reduced the pro-inflammatory factor TNF-α (*p* = 0.000–0.001), IFN-γ (*p* = 0.000–0.002) and IL-6 (*p* = 0.024–0.044) level while improving the anti-inflammatory factor IL-10 level (*p* = 0.009–0.024) compared with the LPS + melatonin group. These responses were reversed by RORα agonists, SR1078. In addition, the addition of the Nrf2 pathway blocker also increased the levels of pro-inflammatory cytokines IL-6 (*p* < 0.001), TNF-α (*p* < 0.001) and IFN-γ (*p* < 0.001) in cell supernatant. However, the results of the pretreatment of B lymphocyte with SR1078 showed the opposite outcomes. These results indicated that melatonin can activate the Nrf2/HO-1 signaling pathway and inhibit the Nfκb pathway by RORα and RORγ nuclear receptors, resulting in anti-inflammatory cytokines’ level enhancing and pro-inflammatory cytokines decreasing.

## 4. Discussion

With the increase in the broiler age, the bursa of Fabricius, which is the primary immunity organ in the chick, will gradually shrink. The bursa volume reached the maximum at sexual maturity and began to degenerate after sexual maturity. Atrophy of the bursa of Fabricius is related to B lymphocyte apoptosis. Previous research found that G→B promoted bursa morphological development and induced B lymphocyte proliferation. However, whether the apoptosis of B lymphocytes in the bursa of Fabricius is affected by different monochromatic light remains to be further explored. In this research, we found that the bursal pro-apoptosis protein Bax and Caspase-3 level were significantly lower in G→B than WW, as well as that the anti-apoptosis protein Bcl-2 level was higher in G→B than WW, indicating G→B could inhibit bursal B lymphocyte apoptosis. Previous research also found G→B could better induce bursal B lymphocyte proliferation [14] and elevate the level of anti-Newcastle disease virus (NDV) and anti-bovine serum albumin (BSA) IgG in plasma compared to WW [3]. These results showed that a combination of green and blue light can promote bursa morphological development as well as improve the humoral immunity of chicks better than white light. However, the bursal Bax and Caspase-3 protein level were the highest in the RR group. Xiong et al. showed that chickens reared under red light have lower T lymphocyte proliferation activity and higher apoptosis protein expression in the thymus, suggesting different light wavelengths have a various effect on the cell vitality in lymphocytes [7,29]. In addition, we observed G→B improved the bursal anti-inflammatory cytokine IL-10 level and decreased the bursal pro-inflammatory IL-6, TNF-α, IFN-γ level as well as enhanced the antioxidant capacity in plasma compared with WW. By contrast, RR improved the plasma pro-inflammatory level and increased lipid peroxidation production of MDA. These results mentioned above indicate that G→B effectively relieves the bursal inflammatory response and protects the body from oxidative stress, thereby inhibiting bursal B lymphocyte apoptosis. However, 660 nm red light improved the IL-6, TNF-α and IFN-γ secretion level to induce the inflammation response, thus leading to the imbalance between the proliferation of the B lymphocyte and apoptosis. Consistent with our results, green light showed the highest IL-2 activity in the spleen during the early growth stage, while the splenic IL-2 activity was the highest under blue light during the later growth stage [30,31]; blue light can effectively reduce the heat stress response in commercial broilers [32], significantly decrease lipid peroxidation and improve the antioxidant activities in the breast and thigh muscles of chicks compared to white light [10].

In addition, G→B improved the plasma melatonin concentration in chickens. Melatonin is a wide-spread hormone, mainly secreted by the pineal gland at night, and its secretion could be affected by environmental light information. For example, the melatonin concentration and release duration in sheep depend on the light cycle, with the highest melatonin concentration in short days (winter) and lowest in long days [33,34]. Interestingly, the Pearson’s correlation analysis indicated that there was a significant negative correlation between the plasma melatonin concentration and apoptosis of B lymphocytes in the bursa of chickens. Previous research reported that melatonin-mediate 560 nm green light inhibited thymus T lymphocyte apoptosis [29], and an exogenous melatonin supplement can inhibit the development and maturation of mouse bone marrow B lymphocytes [35]. These studies indicated that melatonin, as an immune mediator, is crucial for B lymphocyte development. In addition, melatonin has been reported to inhibit pro-inflammatory cytokines IL-8 and TNF-α production in neutrophils [36], suggesting that melatonin helps alleviate acute and chronic inflammatory responses, as well as prevents cell apoptosis through inhibiting the production of pro-inflammatory cytokines [37]. The antioxidant ability of melatonin also accounts for its anti-apoptotic actions on immune cells [37]. Espino et al. found melatonin alleviated aging-induced apoptosis in neutrophils and lymphocytes by neutralizing free radicals and counteracting oxidative stress at the cellular level [38].

Moreover, the functions of melatonin, which exhibits anti-inflammatory, antioxidant and immunity regulation, are often mediated through its binding to the melatonin membrane-bound receptor (Mel1a/Mel1b). In addition to its membrane-bound receptors, studies have found the melatonin binding sites in purified cell nuclear receptors and activated the nuclear receptors in the liver, spleen and thymus [39]. Another study further confirmed the interaction of melatonin and ROR through a co-immunoprecipitation and co-localization assay [40]. In our study, we observed the *RORα* mRNA level was the lowest in the G→B group, whereas it was the highest in the RR group. Additionally, the *RORγ* mRNA level was similar to the RORα results. However, the *RORβ* mRNA level was markedly lower than *RORα* and *RORγ*. The Pearson’s correction results showed there existed a strongly negative correction between the *RORα* or *RORγ* mRNA level and plasma melatonin concentration, indicating melatonin inhibited B lymphocyte apoptosis by negatively regulating the expression of nuclear receptors RORα or RORγ. It has been confirmed in many studies that melatonin can negatively regulate the expression of nuclear receptors. Zhao et al. demonstrated that the regulation of RORα expression by melatonin is dose-dependent, so, as the exogenous melatonin concentration increases, the *RORα* mRNA expression decreases [41]. In addition, Wang et al. proved that melatonin can significantly reduce the mRNA and protein expression levels of RORγ [42]. In in vitro experiments, our results showed that the inhibitory effect of melatonin on B-lymphocyte apoptosis was reversed by SR1078 but enhanced by SR3335. This result corroborates the previous report that melatonin can inhibit apoptosis in mouse Leydig cells via RORα and suppressed Th17 cell differentiation via the inhibition of ROR-γ expression [24].

In our study, we found G→B significantly promoted Nrf2 and its downstream HO-1 protein expression compared with WW, RR, GG, BB and R→B. Melatonin inhibited B lymphocyte apoptosis, and induced pro-apoptosis protein down-regulation was blocked by Nrf2 antagonist ML385, implying the Nrf2 and HO-1 protein may participate in G→B-inhibited B lymphocyte apoptosis. Additionally, melatonin-induced Nrf2 and HO-1activation were blocked by RORα agonist SR1078 and enhanced by RORα antagonist SR3335 or RORγ antagonist GSK2981278, implying that Nrf2 and HO-1 may participate in G→B-inhibited B-lymphocyte apoptosis by RORα/RORγ. Nrf2 (NF-ER-related factor2) is a transcription factor that mediates a broad-based set of adaptive responses to environmental and endogenous stresses, and the absence or inhibition of HO-1 is related to the level of inflammatory response [43]. The Nrf2/HO-1 pathway, which can be activated by the direct binding of the transcription factor to antioxidant response elements, could protect cells from oxidative stress. However, disruption of the Nrf2/HO-1 pathway will exacerbate oxidative stress [44]. Our in vitro results showed that the pretreatment of B lymphocyte with Nrf2 antagonist ML385 not only improved the pro-apoptosis protein expression but also relieved the inhibition of ROS produced by melatonin. Previous research found ROS production resulted in enhancing Nfκb translocation, leading to elevated angiogenic and pro-inflammatory mediators in endometriosis patients [45]. In our study, the protein expression of p-iκb and p-p65 was the lowest in G→B while the highest in RR. In an in vitro experiment, the pretreatment of B lymphocyte with p65 antagonist BAY resulted in reducing Bax and Caspase-3 protein but did not affect the Nrf2 and HO-1 activation. Interestingly, supplementing with ML385 significantly increased the p-iκb protein in the cytoplasm as well as improved the p-p65 protein expression in the nuclear receptor. These results showed that activation of Nrf2/HO-1 can inhibit the activity of Nfκb, thereby inhibiting the inflammatory response, reducing the production of ROS and, ultimately, leading to decreased apoptosis of B lymphocytes. A series of studies have demonstrated that Nrf2 could interact with Nfκb and directly affect the cellular activity of skeletal muscle satellite cells [46], endothelial cells [47] and macrophages [48], supporting our speculation.

Although the modulation of light information on animal immune response has been reported in recent years, there is still a lack of research data on the effect of different monochromatic lights’ combinations on immune function in chicks, especially the neuromodulation mechanism of how the body converts external environmental light stimuli into internal molecular signals to influence a series of physiological changes, including the oxidative stress level and bursal development. In this study, we found that a combination of green and blue light could effectively decrease bursal pro-apoptosis protein Bax, Caspase-3, pro-inflammatory factor TNF-α, IFN-γ, IL-6 and lipid metabolites’ MDA level, increase bursal anti-apoptosis protein Bcl-2, anti-inflammatory factor IL-10 and antioxidant enzyme activity, which promoted bursal development and reduced the stress level. Furthermore, we also explored the signaling pathway of bursal B lymphocyte apoptosis inhibited by melatonin. We considered that our results provide another lighting solution for the rational use of artificial light to reduce the stress levels, enhance health conditions and improve the survival rate of chicks. Therefore, we strongly recommend using 560 nm green light during the early stage (P0–P26) and then transferring to blue light during the later stage (P27–P42) in poultry houses.

## 5. Conclusions

In summary, a combination of green and blue light increased melatonin secretion but inhibited bursal B-lymphocyte apoptosis and the *RORα/RORγ* mRNA level. Melatonin nuclear receptors RORα/RORγ mediate G→B-inhibited bursal B lymphocyte apoptosis via reducing oxidative stress, activating Nrf2/HO-1 and inhibiting the Nfκb pathway in the bursa of chickens. 

## Figures and Tables

**Table 1:** Light parameters.

Items	Light Treatments						
	WW	RR	GG	BB	G→B	B→G	R→B
Light wavelength [nm] (1–26 days)	400–700	660	560	480	560	480	660
Light wavelength [nm] (27–42 days)	400–700	660	560	480	480	560	480
Light intensity [W/m^2^]	0.19	0.19	0.19	0.19	0.19	0.19	0.19
Photoperiod [Light: Dark]	23:1	23:1	23:1	23:1	23:1	23:1	23:1

**Table 2:** Sequences of primers used for RT-PCR.

Gene	Product Size	Primer Sequences (5′–3′)	Accession No.
*RORα*	140	F: TGG GCATACCCCTGAAGGTA<break/>R: CCG ATGCTGGTGTGTAGTCA	XM_413763.2
*RORβ*	270	F: AAA TCG TTG CCA ACA CTG CC<break/>R: AGG TCA ATG ACG TGC CCA TT	NM_205093.1
*RORγ*	90	F: GTG GGGTAATATCGGGAGCG<break/>R: CTT ATCGGGACAACCTGCGT	XM_015280013.1
*GAPDH*	124	F: ATCACAGCCACACAGAAGACG<break/>R: TGACTTTCCCCACAGCCTTA	NM_204305

## Data Availability

Data is contained within the article.

## Abbreviations

Bax	Bcl-2 associated x protein
Bcl-2	B-cell lymphoma-2
BB	Blue light
B→G	A combination of blue and green monochromatic light
Caspases	Cysteinyl aspartate specific proteinase
CAT	Catalase
DAB	3,3′-diaminobenzidine-4HCl
GSH-Px	Glutathione peroxidase
GG	Green light
G→B	A combination of green and blue monochromatic light
HRP	Horseradish-peroxidase
IOD	Integral optical density
INF-γ	Interferon-γ
IL-6	Interleukin-6
IL-10	Interleukin-10
LED	Light Emitting Diode
LPS	Lipopolysaccharide
Mel	Melatonin
Mel1a	Melatonin receptor 1a
Mel1b	Melatonin receptor 1b
Mel1c	Melatonin receptor 1c
MTT	Methyl thiazolyl tetrazolium
MDA	Malondialdehyde
NF-κB	Nuclear factor kappa B
OD	Optical density
PBS	Phosphate-buffered saline
PCNA	Proliferating cell nuclear antigen
RR	Red light
R→B	A combination of red and blue monochromatic light
ROR	Retinoic acid receptor related Orphan Receptor
RORα	Retinoic acid receptor related Orphan Receptor α
RORβ	Retinoic acid receptor related Orphan Receptor β
RORγ	Retinoic acid receptor related Orphan Receptor γ
ROS	Reactive oxygen species
SOD	Superoxide dismutase
SDS	Sodium dodecyl sulfate
T-AOC	Total antioxidant capacity
TNFα	Tumor necrosis factor α
WW	White light
